# 2-(5-{6-[5-(Pyrazin-2-yl)-1*H*-1,2,4-triazol-3-yl]pyridin-2-yl}-1*H*-1,2,4-triazol-3-yl)pyrazine

**DOI:** 10.1107/S1600536811034520

**Published:** 2011-08-27

**Authors:** Zhouqing Xu, Yanchun Sun, Qiang Wang

**Affiliations:** a Department of Physical Chemistry, Henan Polytechnic University, Jiao Zuo 454000, People’s Republic of China; b Department of Medicine, Hebi College of Vocation and Technology, He Bi 458030, People’s Republic of China

## Abstract

In the title mol­ecule, C_17_H_11_N_11_, the five rings are almost coplanar [maxium deviation 0.1949 (1) Å]. The dihedral angles between the two pyrazine rings and the two triazole rings are 1.52 (4) and 2.51 (5)°, respectively. The central pyridine ring forms dihedral angles of 5.57 (1) and 1.71 (1)° with the two triazole rings. The crystal packing consists of a three-dimensional network structure generated by inter­molecular N—H⋯N hydrogen bonds. The crystal structure is further consolidated by π–π stacking [centroid-to-centroid distances 3.599 (10) and 4.769 (13) Å].

## Related literature

For the applications of compounds containing triazole subunits, see: Zhang *et al.* (2010 ▶); Fischer (2007 ▶); Ouellette *et al.* (2007 ▶). For a recent study, see: Xu *et al.* (2011 ▶). For the synthesis, see: Browne (1975 ▶); Klingele & Brooker (2004 ▶). 
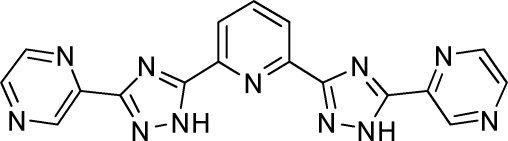


## Experimental

### 

#### Crystal data

C_17_H_11_N_11_
*M* *_r_* = 369.37
Monoclinic, 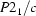

*a* = 10.751 (2) Å
*b* = 13.721 (3) Å
*c* = 11.385 (2) Å
β = 102.57 (3)°
*V* = 1639.2 (6) Å^3^
*Z* = 4
Mo *K*α radiation
μ = 0.10 mm^−1^
*T* = 293 K
0.20 × 0.20 × 0.20 mm

#### Data collection

Bruker SMART APEX diffractometer
Absorption correction: multi-scan (*SADABS*; Sheldrick, 2003 ▶) *T*_min_ = 0.905, *T*_max_ = 1.000
17628 measured reflections
3204 independent reflections
2896 reflections with *I* > 2σ(*I*)
*R*_int_ = 0.048

#### Refinement

*R*[*F* ^2^ > 2σ(*F* ^2^)] = 0.064
*wR*(*F* ^2^) = 0.125
*S* = 1.22
3204 reflections
259 parameters
H atoms treated by a mixture of independent and constrained refinement
Δρ_max_ = 0.17 e Å^−3^
Δρ_min_ = −0.18 e Å^−3^

### 

Data collection: *APEX2* (Bruker, 2003 ▶); cell refinement: *SAINT* (Bruker, 2001 ▶); data reduction: *SAINT*; program(s) used to solve structure: *SHELXS97* (Sheldrick, 2008 ▶); program(s) used to refine structure: *SHELXL97* (Sheldrick, 2008 ▶); molecular graphics: *SHELXTL* (Sheldrick, 2008 ▶); software used to prepare material for publication: *SHELXTL*.

## Supplementary Material

Crystal structure: contains datablock(s) I, global. DOI: 10.1107/S1600536811034520/ng5216sup1.cif

Structure factors: contains datablock(s) I. DOI: 10.1107/S1600536811034520/ng5216Isup2.hkl

Supplementary material file. DOI: 10.1107/S1600536811034520/ng5216Isup3.cml

Additional supplementary materials:  crystallographic information; 3D view; checkCIF report

## Figures and Tables

**Table 1:** Hydrogen-bond geometry (Å, °)

*D*—H⋯*A*	*D*—H	H⋯*A*	*D*⋯*A*	*D*—H⋯*A*
N8—H34⋯N12^i^	0.96 (2)	1.92 (2)	2.817 (3)	155 (2)
N4—H35⋯N13^ii^	0.92 (2)	2.21 (2)	3.113 (3)	167 (2)

Symmetry codes: (i) 
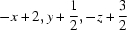
; (ii) 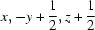.

**Fig. 1.**
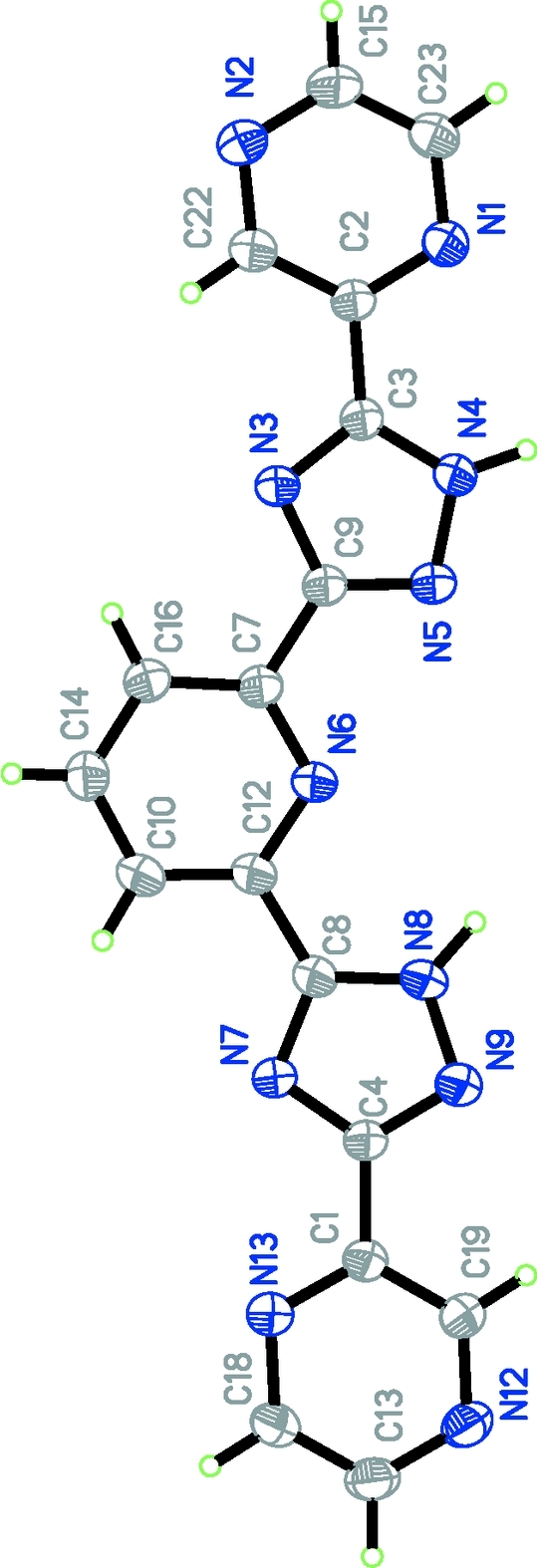
The molecular structure of the title molecule, with the atom-numbering scheme. Displacement ellipsoids are drawn at the 30% probability level.

**Fig. 2.**
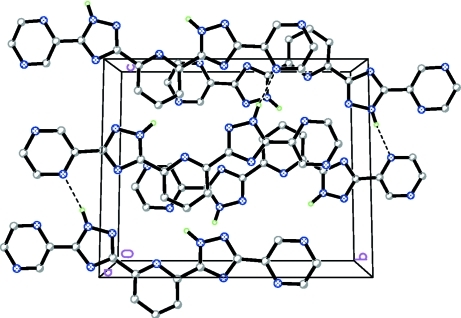
A partial packing diagram of the title compound. Hydrogen bonds are shown as dashed lines, viewed down the a axis.

## supplementary crystallographic information

### Comment

Compounds containing triazole subunits have been intensively studied owing to diverse biological properties and also to the ease of complexation (Zhang *et al.*, 2010; Fischer, 2007; Ouellette *et al.*, 2007). In continuation with a recent study (Xu *et al.*, 2011), we report the title compound (Scheme I), 2-(5-(6-(5-(pyrazin-2-yl)-1*H*-1,2,4-triazol-3-yl)pyridin-2-yl)-1*H*-1,2,4-triazol-3-yl)pyrazine.

The five rings are almost coplanar (maxium deviation = 0.1949 (1) Å). The dihedral angles between the two pyrazine rings and the two triazole rings are 1.52 (4) and 2.51 (5)°, respectively. The central pyridine ring forms dihedral angles of 5.57 (1) and 1.71 (1)° with the two triazole rings (Fig. 1). The crystal packing consists of a three-dimensional net structure generated by intermolecular N—H···N hydrogen bonds. The crystal structure is further consolidated by π–π stacking [centroid–centroid distances = 3.599 (10) and 4.769 (13) Å].

### Experimental

2-(5-(6-(5-(pyrazin-2-yl)-1*H*-1,2,4-triazol-3-yl)pyridin-2-yl)-1*H*-1,2,4-triazol-3-yl)pyrazine was prepared according to Browne (1975) and Klingele & Brooker (2004). The crystals suitable for crystallographic analysis were grown by recrystallization from DMF and ethanol solution as colorless prism.

### Refinement

N-bound H atoms were located in a difference map and refined freely. C-bound H atoms were positioned geometrically (C—H = 0.94 Å) and were constrained in a riding motion approximation with *U*_iso_(H) = 1.2*U*_eq_(C).

### Crystal data

**Table:**

C_17_H_11_N_11_	*F*(000) = 760
*M**_r_* = 369.37	*D*_x_ = 1.497 Mg m^−^^3^
Monoclinic, *P*2_1_/*c*	Mo *K*α radiation, λ = 0.71073 Å
Hall symbol: -P 2ybc	Cell parameters from 4186 reflections
*a* = 10.751 (2) Å	θ = 1.9–27.1°
*b* = 13.721 (3) Å	µ = 0.10 mm^−^^1^
*c* = 11.385 (2) Å	*T* = 293 K
β = 102.57 (3)°	Prism, colourless
*V* = 1639.2 (6) Å^3^	0.20 × 0.20 × 0.20 mm
*Z* = 4	

### Data collection

**Table:**

Bruker SMART APEX diffractometer	3204 independent reflections
Radiation source: fine-focus sealed tube	2896 reflections with *I* > 2σ(*I*)
graphite	*R*_int_ = 0.048
ω scans	θ_max_ = 26.0°, θ_min_ = 2.4°
Absorption correction: multi-scan (*SADABS*; Sheldrick, 2003)	*h* = −13→13
*T*_min_ = 0.905, *T*_max_ = 1.000	*k* = −16→16
17628 measured reflections	*l* = −13→14

### Refinement

**Table:**

Refinement on *F*^2^	Primary atom site location: structure-invariant direct methods
Least-squares matrix: full	Secondary atom site location: difference Fourier map
*R*[*F*^2^ > 2σ(*F*^2^)] = 0.064	Hydrogen site location: inferred from neighbouring sites
*wR*(*F*^2^) = 0.125	H atoms treated by a mixture of independent and constrained refinement
*S* = 1.22	*w* = 1/[σ^2^(*F*_o_^2^) + (0.042*P*)^2^ + 0.4894*P*] where *P* = (*F*_o_^2^ + 2*F*_c_^2^)/3
3204 reflections	(Δ/σ)_max_ < 0.001
259 parameters	Δρ_max_ = 0.17 e Å^−^^3^
0 restraints	Δρ_min_ = −0.18 e Å^−^^3^

### Special details

**Table:**

Geometry. All e.s.d.'s (except the e.s.d. in the dihedral angle between two l.s. planes) are estimated using the full covariance matrix. The cell e.s.d.'s are taken into account individually in the estimation of e.s.d.'s in distances, angles and torsion angles; correlations between e.s.d.'s in cell parameters are only used when they are defined by crystal symmetry. An approximate (isotropic) treatment of cell e.s.d.'s is used for estimating e.s.d.'s involving l.s. planes.
Refinement. Refinement of *F*^2^ against ALL reflections. The weighted *R*-factor *wR* and goodness of fit *S* are based on *F*^2^, conventional *R*-factors *R* are based on *F*, with *F* set to zero for negative *F*^2^. The threshold expression of *F*^2^ > σ(*F*^2^) is used only for calculating *R*-factors(gt) *etc*. and is not relevant to the choice of reflections for refinement. *R*-factors based on *F*^2^ are statistically about twice as large as those based on *F*, and *R*-factors based on ALL data will be even larger.

### Fractional atomic coordinates and isotropic or equivalent isotropic displacement parameters (Å^2^)

**Table:**

	*x*	*y*	*z*	*U*_iso_*/*U*_eq_	
N7	0.80331 (17)	0.04695 (12)	0.48120 (15)	0.0328 (4)	
N3	0.65636 (17)	0.55996 (12)	0.51910 (15)	0.0338 (4)	
N6	0.74831 (16)	0.30212 (12)	0.54161 (15)	0.0324 (4)	
N1	0.67651 (19)	0.77043 (13)	0.71611 (16)	0.0429 (5)	
N4	0.73685 (19)	0.56738 (13)	0.71244 (16)	0.0391 (5)	
H35	0.764 (2)	0.5883 (17)	0.791 (2)	0.047*	
N5	0.75302 (19)	0.47317 (13)	0.68211 (16)	0.0412 (5)	
C19	0.9825 (2)	−0.15553 (16)	0.66349 (19)	0.0382 (5)	
H19	1.0142	−0.1196	0.7328	0.046*	
C1	0.90635 (19)	−0.10896 (14)	0.56482 (18)	0.0308 (5)	
N9	0.91344 (18)	0.04313 (13)	0.67510 (16)	0.0401 (5)	
C2	0.64501 (19)	0.72112 (15)	0.61304 (18)	0.0308 (5)	
C3	0.6795 (2)	0.61778 (15)	0.61474 (18)	0.0311 (5)	
N8	0.86523 (18)	0.13308 (13)	0.64512 (17)	0.0387 (5)	
H34	0.887 (2)	0.1853 (17)	0.702 (2)	0.046*	
C4	0.87349 (19)	−0.00581 (14)	0.57292 (18)	0.0307 (5)	
N13	0.86310 (17)	−0.15592 (13)	0.46165 (16)	0.0365 (4)	
N12	1.01117 (18)	−0.24929 (13)	0.66175 (17)	0.0412 (5)	
C7	0.6963 (2)	0.38491 (14)	0.48938 (18)	0.0311 (5)	
C8	0.8012 (2)	0.13480 (15)	0.52990 (18)	0.0307 (5)	
C9	0.7024 (2)	0.47236 (14)	0.56489 (18)	0.0318 (5)	
C10	0.6893 (2)	0.22266 (16)	0.34824 (19)	0.0368 (5)	
H10	0.6895	0.1666	0.3023	0.044*	
C23	0.6418 (3)	0.86407 (17)	0.7106 (2)	0.0495 (6)	
H23	0.6623	0.9015	0.7802	0.059*	
C12	0.74258 (19)	0.22300 (14)	0.47064 (18)	0.0306 (5)	
C18	0.8945 (2)	−0.25062 (16)	0.4604 (2)	0.0406 (6)	
H18	0.8673	−0.2861	0.3899	0.049*	
N2	0.54644 (19)	0.85860 (14)	0.50318 (17)	0.0447 (5)	
C13	0.9652 (2)	−0.29676 (16)	0.5592 (2)	0.0410 (6)	
H13	0.9816	−0.3631	0.5548	0.049*	
C14	0.6359 (2)	0.30832 (16)	0.2967 (2)	0.0393 (5)	
H14	0.5992	0.3110	0.2149	0.047*	
C15	0.5771 (2)	0.90720 (17)	0.6064 (2)	0.0468 (6)	
H15	0.5541	0.9724	0.6081	0.056*	
C16	0.6376 (2)	0.38988 (15)	0.36799 (19)	0.0363 (5)	
H16	0.5999	0.4476	0.3352	0.044*	
C22	0.5812 (2)	0.76535 (16)	0.5078 (2)	0.0396 (5)	
H22	0.5619	0.7285	0.4377	0.048*	

### Atomic displacement parameters (Å^2^)

**Table:**

	*U*^11^	*U*^22^	*U*^33^	*U*^12^	*U*^13^	*U*^23^
N7	0.0419 (10)	0.0238 (9)	0.0304 (9)	0.0030 (8)	0.0026 (8)	0.0000 (7)
N3	0.0461 (11)	0.0226 (9)	0.0304 (9)	0.0026 (8)	0.0033 (8)	−0.0003 (7)
N6	0.0418 (10)	0.0226 (9)	0.0314 (9)	0.0008 (8)	0.0049 (8)	−0.0015 (7)
N1	0.0583 (12)	0.0295 (10)	0.0348 (10)	0.0045 (9)	−0.0030 (9)	−0.0043 (8)
N4	0.0593 (13)	0.0247 (10)	0.0288 (10)	0.0048 (9)	−0.0001 (9)	−0.0029 (8)
N5	0.0619 (13)	0.0242 (10)	0.0334 (10)	0.0060 (9)	0.0013 (9)	−0.0006 (8)
C19	0.0474 (13)	0.0293 (12)	0.0336 (12)	−0.0008 (10)	−0.0007 (10)	0.0024 (9)
C1	0.0340 (11)	0.0257 (11)	0.0312 (11)	−0.0026 (9)	0.0034 (9)	−0.0002 (8)
N9	0.0505 (11)	0.0277 (10)	0.0363 (10)	0.0023 (8)	−0.0033 (9)	−0.0028 (8)
C2	0.0363 (11)	0.0246 (11)	0.0305 (11)	0.0020 (9)	0.0048 (9)	0.0004 (8)
C3	0.0376 (11)	0.0258 (11)	0.0281 (11)	0.0016 (9)	0.0032 (9)	0.0000 (8)
N8	0.0518 (12)	0.0241 (10)	0.0359 (11)	0.0015 (8)	0.0002 (9)	−0.0050 (8)
C4	0.0373 (12)	0.0230 (10)	0.0300 (11)	−0.0009 (9)	0.0032 (9)	0.0004 (8)
N13	0.0445 (11)	0.0278 (10)	0.0345 (10)	0.0011 (8)	0.0025 (8)	−0.0006 (8)
N12	0.0453 (11)	0.0314 (11)	0.0427 (11)	0.0023 (9)	0.0003 (9)	0.0073 (9)
C7	0.0372 (11)	0.0244 (11)	0.0307 (11)	−0.0010 (9)	0.0052 (9)	0.0010 (8)
C8	0.0367 (11)	0.0248 (10)	0.0299 (11)	−0.0012 (9)	0.0056 (9)	−0.0023 (8)
C9	0.0401 (12)	0.0230 (10)	0.0302 (11)	0.0009 (9)	0.0030 (9)	0.0005 (8)
C10	0.0468 (13)	0.0270 (12)	0.0355 (12)	0.0001 (10)	0.0068 (10)	−0.0055 (9)
C23	0.0717 (17)	0.0291 (13)	0.0412 (13)	0.0063 (12)	−0.0017 (12)	−0.0085 (10)
C12	0.0358 (11)	0.0239 (11)	0.0317 (11)	−0.0007 (9)	0.0065 (9)	−0.0023 (8)
C18	0.0488 (13)	0.0291 (12)	0.0399 (13)	0.0030 (10)	0.0004 (11)	−0.0065 (10)
N2	0.0603 (13)	0.0323 (11)	0.0390 (11)	0.0109 (9)	0.0051 (10)	0.0030 (8)
C13	0.0437 (13)	0.0267 (12)	0.0493 (14)	0.0019 (10)	0.0031 (11)	0.0021 (10)
C14	0.0501 (14)	0.0348 (13)	0.0297 (12)	−0.0002 (10)	0.0018 (10)	0.0000 (9)
C15	0.0606 (16)	0.0272 (12)	0.0493 (14)	0.0089 (11)	0.0049 (12)	−0.0014 (11)
C16	0.0470 (13)	0.0261 (11)	0.0333 (12)	0.0014 (10)	0.0031 (10)	0.0022 (9)
C22	0.0515 (14)	0.0296 (12)	0.0354 (12)	0.0064 (10)	0.0041 (11)	−0.0025 (9)

### Geometric parameters (Å, °)

**Table:**

N7—C8	1.329 (2)	N8—H34	0.96 (2)
N7—C4	1.357 (3)	N13—C18	1.343 (3)
N3—C3	1.326 (2)	N12—C13	1.334 (3)
N3—C9	1.360 (2)	C7—C16	1.390 (3)
N6—C7	1.346 (2)	C7—C9	1.469 (3)
N6—C12	1.347 (2)	C8—C12	1.460 (3)
N1—C2	1.333 (3)	C10—C14	1.381 (3)
N1—C23	1.336 (3)	C10—C12	1.387 (3)
N4—C3	1.340 (3)	C10—H10	0.9300
N4—N5	1.359 (2)	C23—C15	1.372 (3)
N4—H35	0.92 (2)	C23—H23	0.9300
N5—C9	1.327 (3)	C18—C13	1.368 (3)
C19—N12	1.324 (3)	C18—H18	0.9300
C19—C1	1.393 (3)	N2—C15	1.329 (3)
C19—H19	0.9300	N2—C22	1.331 (3)
C1—N13	1.332 (3)	C13—H13	0.9300
C1—C4	1.467 (3)	C14—C16	1.380 (3)
N9—C4	1.331 (3)	C14—H14	0.9300
N9—N8	1.353 (2)	C15—H15	0.9300
C2—C22	1.384 (3)	C16—H16	0.9300
C2—C3	1.465 (3)	C22—H22	0.9300
N8—C8	1.342 (3)		
			
C8—N7—C4	102.71 (16)	N7—C8—C12	127.14 (18)
C3—N3—C9	103.07 (16)	N8—C8—C12	122.96 (18)
C7—N6—C12	117.06 (17)	N5—C9—N3	114.43 (18)
C2—N1—C23	115.61 (19)	N5—C9—C7	123.46 (18)
C3—N4—N5	109.94 (17)	N3—C9—C7	122.10 (18)
C3—N4—H35	129.4 (15)	C14—C10—C12	117.8 (2)
N5—N4—H35	120.7 (15)	C14—C10—H10	121.1
C9—N5—N4	102.49 (17)	C12—C10—H10	121.1
N12—C19—C1	122.1 (2)	N1—C23—C15	122.6 (2)
N12—C19—H19	118.9	N1—C23—H23	118.7
C1—C19—H19	118.9	C15—C23—H23	118.7
N13—C1—C19	121.37 (19)	N6—C12—C10	124.10 (19)
N13—C1—C4	118.67 (18)	N6—C12—C8	115.60 (18)
C19—C1—C4	119.95 (18)	C10—C12—C8	120.26 (18)
C4—N9—N8	101.92 (17)	N13—C18—C13	122.4 (2)
N1—C2—C22	121.49 (19)	N13—C18—H18	118.8
N1—C2—C3	117.69 (18)	C13—C18—H18	118.8
C22—C2—C3	120.82 (18)	C15—N2—C22	115.70 (19)
N3—C3—N4	110.06 (18)	N12—C13—C18	121.8 (2)
N3—C3—C2	124.49 (18)	N12—C13—H13	119.1
N4—C3—C2	125.44 (18)	C18—C13—H13	119.1
C8—N8—N9	110.45 (17)	C16—C14—C10	119.3 (2)
C8—N8—H34	129.9 (14)	C16—C14—H14	120.4
N9—N8—H34	119.3 (14)	C10—C14—H14	120.4
N9—C4—N7	115.03 (18)	N2—C15—C23	122.0 (2)
N9—C4—C1	120.37 (18)	N2—C15—H15	119.0
N7—C4—C1	124.60 (17)	C23—C15—H15	119.0
C1—N13—C18	115.89 (18)	C14—C16—C7	119.3 (2)
C19—N12—C13	116.37 (19)	C14—C16—H16	120.3
N6—C7—C16	122.38 (19)	C7—C16—H16	120.3
N6—C7—C9	118.00 (18)	N2—C22—C2	122.5 (2)
C16—C7—C9	119.62 (18)	N2—C22—H22	118.7
N7—C8—N8	109.88 (18)	C2—C22—H22	118.7

### Hydrogen-bond geometry (Å, °)

**Table:**

*D*—H···*A*	*D*—H	H···*A*	*D*···*A*	*D*—H···*A*
N8—H34···N12^i^	0.96 (2)	1.92 (2)	2.817 (3)	155 (2)
N4—H35···N13^ii^	0.92 (2)	2.21 (2)	3.113 (3)	167 (2)

Symmetry codes: (i) −*x*+2, *y*+1/2, −*z*+3/2; (ii) *x*, −*y*+1/2, *z*+1/2.
